# The Korea Acute Myocardial Infarction Registry: Two Decades of AMI Care in Korea

**DOI:** 10.1016/j.jacasi.2026.07.043

**Published:** 2026-10-06

**Authors:** Doo Sun Sim, Myung Ho Jeong

**Affiliations:** a Department of Cardiology, Chonnam National University Hospital, Chonnam National University Medical School, Gwangju, Korea; b Department of Cardiology, Gwangju Veterans Hospital, Gwangju, Korea

**Keywords:** dyslipidemias, percutaneous coronary intervention, platelet aggregation inhibitors

**Figure:**
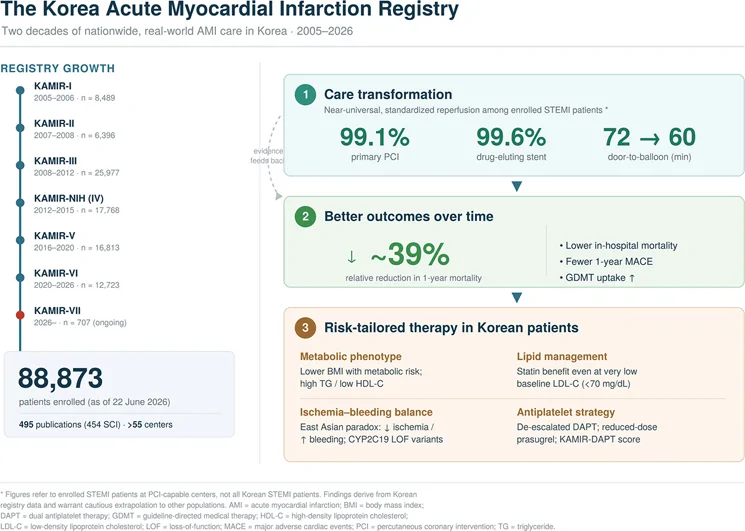

Cardiovascular disease trends have evolved heterogeneously across regions. Although several Western countries have reported declining mortality from coronary artery disease over recent decades, East Asian countries continue to experience a substantial and, in some settings, increasing burden of ischemic heart disease.^1^ These regional differences underscore the importance of locally derived data to inform clinical decision-making and health policy. The Korea Acute Myocardial Infarction Registry (KAMIR), established in 2005, represents the first nationwide, prospective, multicenter registry of acute myocardial infarction (AMI) in Korea.^2^ Over the past 20 years, KAMIR has generated large-scale, real-world evidence encompassing epidemiology, treatment strategies, and longitudinal outcomes in Korean patients with AMI. The registry has provided critical insights into clinical characteristics of Korean patients, including differences in metabolic risk profiles and antithrombotic response patterns, while documenting substantial improvements in system-level care and clinical outcomes.

This article summarizes the 20-year experience of KAMIR, emphasizing: 1) epidemiologic transitions; 2) system-level outcome improvements associated with implementation of evidence-based therapies; 3) distinctive Korean and East Asian risk profiles and therapeutic response patterns; 4) the position of KAMIR among other Asian registries; and 5) the influence of registry-derived evidence on guideline development, together with a candid appraisal of the limitations of registry-based evidence.

Rather than cataloging the many individual analyses KAMIR has produced, this Landscape article offers a system-level, comparative, and limitation-aware synthesis that complements the comprehensive 20-year clinical review^2^ with an international perspective.

## The Korea Acute Myocardial Infarction Registry

KAMIR was initiated in November 2005 to systematically collect data on patients with AMI across Korea.^2^ It became the first prospective, multicenter AMI registry in the country, enrolling patients from over 50 hospitals (both community and academic centers) with percutaneous coronary intervention (PCI) capability. Through 6 sequential phases, cumulative enrollment exceeded 70,000 patients by 2018 and approximately 87,000 by mid-2025.^2^ With the ongoing seventh phase (KAMIR-VII), cumulative enrollment reached 88,873 patients with more than 495 publications (454 indexed in Science Citation Index) as of 22 June 2026. Its comprehensive case report forms capture patient demographics, risk factors, clinical presentation, management strategies, and outcomes, with standardized definitions to ensure data quality. The phased evolution and cumulative enrollment of the registry are illustrated in the Figure 1.

**Figure 1:**
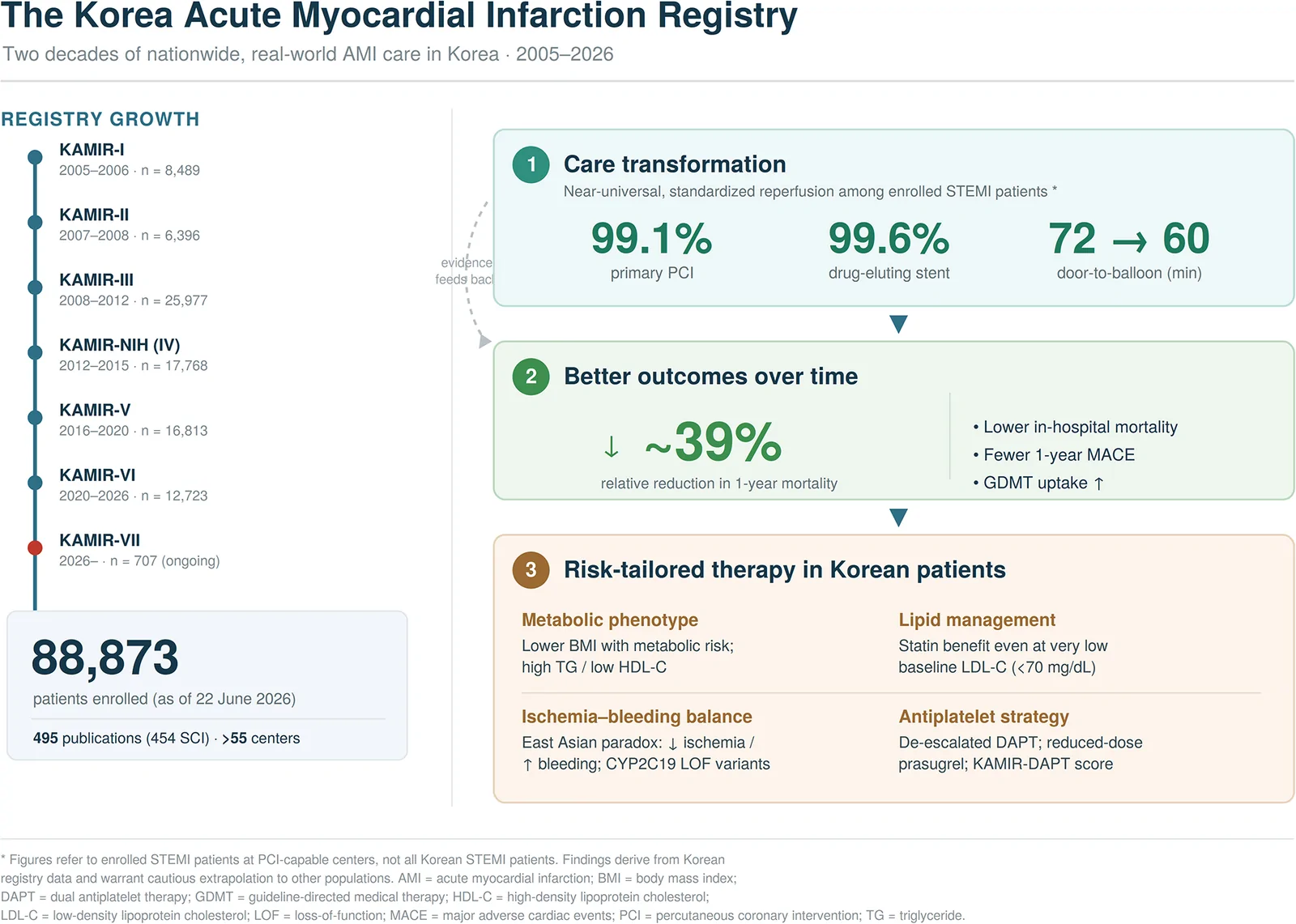
Two Decades of KAMIR: Growth, Outcomes, and Tailored Therapy The left traces the growth of the KAMIR through its sequential phases (KAMIR-I to KAMIR-VII), reaching 88,873 enrolled patients and 495 publications across more than 55 centers. The 3 numbered strips on the right show the registry-driven sequence: 1) care transformation, with near-universal primary percutaneous coronary intervention (PCI); drug-eluting stenting, and shortened door-to-balloon time; 2) better outcomes, including an approximately 39% relative reduction in 1-year mortality; and 3) risk-tailored therapy in Korean patients. The dashed arrow denotes evidence feeding back to the registry. The asterisk indicates figures among enrolled patients with STEMI at PCI-capable centers. AMI = acute myocardial infarction; BMI = body mass index; GDMT = guideline-directed medical therapy; HDL-C = high-density lipoprotein cholesterol; KAMIR-DAPT = Korea Acute Myocardial Infarction Registry–Dual Antiplatelet Therapy; KAMIR-NIH = Korea Acute Myocardial Infarction Registry–National Institutes of Health; LDL-C = low-density lipoprotein cholesterol; LOF = loss of function; MACE = major adverse cardiac events; SCI = Science Citation Index; STEMI = ST-segment elevation myocardial infarction; TG = triglycerides.

Importantly, KAMIR provides insight into how Korean patients with AMI may differ from their Western counterparts in epidemiology and treatment response. Its scale and longitudinal design distinguish it from single-center or short-term observational studies. Because enrollment is restricted to PCI-capable centers, however, the cohort is best understood as real-world rather than population-representative; this and other limitations are discussed below.

As this article is a narrative review based on previously published, deidentified, aggregate registry data, it does not constitute human-subjects research, and institutional review board approval was not applicable.

## Epidemiologic Transition and Outcome Improvements

### Shift from STEMI to NSTEMI

KAMIR has documented a marked transition in the spectrum of AMI presentation. In 2005, ST-segment elevation myocardial infarction (STEMI) accounted for 64.3% of AMI cases in KAMIR; by 2018, this proportion had decreased to 48.4%, with crossover to non-STEMI (NSTEMI) occurring around 2012.^3^ This crossover parallels trends observed in Western populations and likely reflects improved detection of NSTEMI through high-sensitivity cardiac troponin assays and enhanced risk factor management.^2^

### Aging population

The mean age of Korean patients with AMI has progressively increased, reflecting population aging and a growing proportion of elderly patients in contemporary cohorts.^3^ Despite higher baseline risk, registry analyses demonstrate that older patients derive meaningful benefit from guideline-directed therapies, including primary PCI, when clinically appropriate.^2^ These findings support the extension of evidence-based interventions to elderly patients rather than therapeutic conservatism based solely on age.

### System-level improvements

The most compelling contribution of KAMIR lies in its documentation of outcome improvements over time. A registry-wide trend analysis showed an approximately 39% relative reduction in 1-year mortality.^2^^,^^3^ The corresponding absolute reduction was more modest, and part of the improvement likely reflects secular trends, earlier and milder presentations, and lead-time effects of high-sensitivity troponin rather than specific interventions alone. By 2018, among enrolled patients with STEMI at PCI-capable centers, primary PCI was performed in 99.1% of cases, and drug-eluting stents were implanted in 99.6%.^3^ Door-to-balloon times were significantly shortened nationwide, from a median of 72 minutes in 2008 to 60 minutes in 2018,^3^ with shorter reperfusion delays independently associated with lower mortality.^2^ Concomitantly, adherence to guideline-directed medical therapy—dual antiplatelet therapy (DAPT), high-intensity statin, β-blocker, and renin–angiotensin system inhibitor—improved substantially.^3^ Collectively, these coordinated system-level changes accompanied sustained reductions in in-hospital mortality and 1-year major adverse cardiac events.^2^^,^^3^

## Distinct Risk Profiles in Korean Patients

KAMIR has identified several features that distinguish Korean patients with AMI from Western cohorts. These observations derive from Korean data and should be extrapolated to other populations with caution.

### Metabolic phenotype and dyslipidemia

Although hypertension and diabetes mellitus remain prevalent, Korean patients with AMI often present with relatively lower body mass index despite increased metabolic risk.^4^ Diabetes prevalence rose over time, reaching 41.8% by 2018.^3^ Dyslipidemia frequently manifests as elevated triglycerides and reduced high-density lipoprotein cholesterol rather than markedly elevated low-density lipoprotein cholesterol (LDL-C),^3^ and high triglyceride-to-high-density lipoprotein ratios have been associated with worse outcomes.^2^ Notably, KAMIR demonstrated the benefit of statin therapy even at very low baseline LDL-C (<70 mg/dL),^2^ supporting intensive lipid lowering irrespective of baseline LDL-C and cautioning against reliance on LDL-C thresholds alone.

## The East Asian Paradox in Antiplatelet Therapy

The East Asian Paradox refers to the observation that, on equivalent intensities of antiplatelet therapy, East Asian patients tend to experience lower ischemic event rates yet higher bleeding rates than predominantly European or North American patients.^1^ This pattern derives chiefly from observational and registry comparisons with heterogeneous DAPT regimens, is not universal, and remains debated.

Genetic background partly contributes to this divergence. East Asian populations exhibit a higher prevalence of CYP2C19 loss-of-function alleles, which influence clopidogrel metabolism and on-treatment platelet reactivity.^1^ Variation in transporter and esterase genes (eg, ABCB1, CES1) and nongenetic factors—lower body weight, dietary and comorbidity patterns—also contribute, so the paradox may partly reflect differential baseline risk rather than biology alone. In KAMIR analyses, escalation to more potent P2Y12 inhibitors did not consistently yield proportional ischemic benefit under the specific comparisons examined; standard-dose ticagrelor or prasugrel did not significantly reduce ischemic endpoints vs clopidogrel but were associated with more bleeding.^2^ Although potent agents reduced ischemic events in predominantly Western trials and may benefit selected higher-risk subgroups,^1^ such benefit was not consistently observed in these registry comparisons, which are further limited by nonrandomized dosing and residual confounding.^2^^,^^5^

In a collaborative analysis with the Japanese registry, a reduced-dose prasugrel regimen was associated with less in-hospital major bleeding, while 12-month major adverse cardiac events were similar between groups.^6^ This data set is observational and nonrandomized, and its findings require confirmation. To formalize risk-adapted decisions, the KAMIR-DAPT score integrates ischemic and bleeding predictors and improves discrimination in Korean cohorts.^7^ These observations informed regional consensus emphasizing individualized antiplatelet strategy rather than uniform Western intensification.^1^

## Procedural and Therapeutic Optimization

KAMIR has also informed procedural and adjunctive strategy. In hemodynamically stable multivessel STEMI, staged complete revascularization was associated with better long-term outcomes than culprit-only PCI, consistent with randomized evidence; in cardiogenic shock, immediate multivessel PCI conferred no short-term benefit.^2^^,^^8^ Intravascular imaging guidance was associated with lower target-lesion failure.^2^ Discharge β-blocker and renin–angiotensin system inhibition were linked to better outcomes chiefly in patients with reduced systolic function, supporting risk-stratified guideline-directed medical therapy,^2^ and registry-derived machine-learning models improved outcome discrimination vs conventional risk scores.^2^

## KAMIR in the Context of Other Asian Registries

KAMIR is one of several major Asian cardiovascular registries, each with complementary strengths. Japan's J-PCI (Japanese PCI) is a very large procedure-focused registry capturing nationwide PCI volume and outcomes, whereas China's Improving Care for Cardiovascular Disease in China–Acute Coronary Syndrome program is a hospital-based quality-improvement registry for acute coronary syndrome. The American College of Cardiology's Global Heart Attack Treatment Initiative targets lower- and middle-income settings. Relative to these, KAMIR's distinguishing feature is its sustained, multiphase longitudinal design spanning the full AMI spectrum. Direct cross-registry comparison is constrained by differences in case definition, enrollment, and follow-up; nonetheless, KAMIR's phased methodology may serve as a practical reference for emerging registries such as Global Heart Attack Treatment Initiative. These observations are descriptive and not based on harmonized data.

## Influence on Guidelines and Practice in Korea

Evidence generated by KAMIR has informed Korean guideline development. The 2021 Korean Society of Myocardial Infarction Expert Consensus on Revascularization reflects registry-based evidence supporting primary PCI and refined revascularization strategy,^9^ followed by the 2024 Korean Society of Myocardial Infarction/National Evidence-Based Healthcare Collaborating Agency Guideline for the Pharmacotherapy of Acute Coronary Syndromes, which incorporates registry-derived findings on intensive statin therapy and individualized DAPT.^10^ At a regional level, antiplatelet consensus statements explicitly acknowledge the East Asian paradox and emphasize risk-tailored strategies.^1^ Rather than altering specific non-Korean national guidelines, KAMIR contributes to a shared regional evidence base.

### Study limitations

First, enrollment is restricted to PCI-capable hospitals, so patients managed at non-PCI centers and those who die before reaching care are under-represented, biasing the cohort toward survivors reaching advanced care and likely toward larger, more urban institutions; the participating hospitals are therefore not a population-based sample of Korean practice. Consequently, figures such as the 99.1% primary PCI rate describe enrolled patients with STEMI at PCI-capable centers rather than all Korean patients with STEMI. Second, like all registries, KAMIR is subject to loss to follow-up and variable completeness of long-term events, and not every variable is independently audited despite central monitoring. Third, observed outcome improvements are associational; secular trends, a shift toward milder or earlier-detected cases, and lead-time bias contribute alongside specific interventions. Fourth, most evidence is Korea-specific and may not generalize across East or South Asia. Finally, some KAMIR findings—benefit of statins at very low LDL-C and the antithrombotic paradox—challenged prevailing assumptions and remain debated. Priorities to address these gaps include linkage with national administrative and mortality databases to capture out-of-hospital events, prospective randomization embedded within the registry, and standardized cross-Asian data harmonization.

## Conclusions

Over 2 decades, KAMIR has advanced understanding of AMI in Korea. The registry documented major epidemiologic transitions, accompanied by near-universal primary PCI and contemporary drug-eluting stents among enrolled patients, and tracked a substantial relative reduction in 1-year mortality. It characterized distinctive risk profiles and differential antiplatelet responses and supported individualized therapeutic strategies. By linking observational evidence to guideline refinement while remaining mindful of its limitations, KAMIR exemplifies how a sustained national registry can function as a quality-improvement platform whose methodological lessons may inform emerging registries across Asia.

### Ethics Statement

This article is a narrative review based on previously published, deidentified, aggregate registry data, and does not constitute human-subjects research. Accordingly, institutional review board approval was not applicable.

## Funding Support and Author Disclosures

This study was supported by a grant (BCRI-26080) of Chonnam National University Hospital Biomedical Research Institute. The authors have reported that they have no relationships relevant to the contents of this paper to disclose.
